# Applying Trauma-Informed Practices to the Care of Refugee and Immigrant Youth: 10 Clinical Pearls

**DOI:** 10.3390/children6080094

**Published:** 2019-08-20

**Authors:** Kathleen K. Miller, Calla R. Brown, Maura Shramko, Maria Veronica Svetaz

**Affiliations:** Department of General Pediatrics and Adolescent Health, University of Minnesota, 717 Delaware St SE, Minneapolis, MN 55414, USA

**Keywords:** youth, refugee, immigrant, trauma, health care

## Abstract

Immigrant and refugee youth have higher rates of trauma than youth who are not transnational. While youth are incredibly resilient, trauma and toxic stress can result in poor health outcomes that persist throughout life. However, clinical interventions can promote resilience and decrease the negative impact of trauma. This article will review the principles of trauma-informed care and its application for the care of immigrant and refugee youth and their families by sharing concrete and feasible strategies for primary care providers and systems.

## 1. Introduction

The past decades have seen increasing rates of migration for children and families across international borders. In 2017, as many as 30 million children and youth under the age of 18 were forcibly displaced. Seventeen million of these youth experienced violence or conflict in their home country, and approximately 13 million were eligible for refugee status [1]. Many of these children experienced significant trauma prior to migration, through civil war or unrest, destructive effects of climate change, gang or drug related violence, or poverty [2]. Children are also vulnerable during the migration journey and may be the victims of physical or sexual abuse, unsafe travel conditions, separation from family members, and trafficking [2]. Furthermore, upon arrival in new countries, immigrant youth and families may experience xenophobia and discrimination [3,4,5]. While these youth possess enormous resilience and strength, the experiences of repeated and prolonged exposure to trauma place them at risk for adverse health outcomes. Trauma and toxic stress are associated with higher rates of depression, anxiety, post-traumatic stress syndrome (PTSD), heart disease, metabolic syndrome, and early death [6,7,8].

No matter where a clinician is practicing in the world, taking care of children and youth who are immigrants or refugees means caring for children and youth that may have experienced trauma. It is well known that health care centers and primary care providers play a crucial role in promoting resiliency and mitigating the negative outcomes of trauma for immigrant and refugee families [9,10,11]. The American Academy of Pediatrics recommends utilizing trauma-informed care (TIC) practices when working with immigrant and refugee families [12]. However, it can be challenging to implement these practices without a clear framework and tangible strategies. This article will provide a brief and practical review of TIC and 10 concrete and feasible clinical pearls for promoting a trauma-informed practice with this population.

## 2. What Is Trauma-Informed Care?

Trauma is defined as “an event, series of events, or set of circumstances that is experienced by an individual as physically or emotionally harmful or life-threatening and that has lasting adverse effects on the individual’s functioning and mental, physical, social, emotional, or spiritual well-being” [13]. In the context of pediatric care, TIC is an approach that recognizes the pervasive impact of trauma on children’s development and health (as well more broadly throughout systems of service delivery), applies this knowledge of trauma and its consequence into practice, and actively seeks to prevent re-traumatization [13,14,15,16]. It is often considered a lens through which to view and interpret child health and outcomes, rather than a specific set of directives. Principles of TIC include promoting physical and psychological safety for patients, building trusting relationships with patients and families, providing peer support, collaborating with patients and families, supporting and fostering agency, and promoting intersectionality [9,12]. Both the American Academy of Pediatrics and the Budapest Declaration on the Rights, Health, and Well-Being of Children and Youth on the Move have identified TIC as a best practice in the care of immigrant and refugee youth [8,13,17].

TIC can be challenging to implement as it is a philosophical approach to care that often encompasses multiple systems and disciplines that were not designed to acknowledge or address experiences of trauma. In this article, we offer 10 clinical pearls to serve as suggestions for starting points to promote TIC for immigrant and refugee families. While not comprehensive, these steps include concrete steps for primary care providers and clinics to support the implementation of TIC.

## 3. Ten Clinical Pearls for Applying a Trauma-Informed Approach to Care for Refugee and Immigrant Youth

### 3.1. Practice a Strengths-Based Approach to Care

Although many immigrant and refugee youth have experienced adversity and hardship, first and foremost, they are people who have also drawn on significant internal and external strengths to have survived their past experiences. A critical first step in TIC is to recognize and treat immigrant and refugee children as such. Children are inherently resilient, and immigrant and refugee youth may additionally draw on community and ethnic resiliency [18]. TIC acknowledges and promotes their strengths, resiliency, and capacity to heal from trauma [11,12]. In contrast to a deficit-focused approach, a strengths-based approach focuses on growth and development and recognizes that acknowledging strengths can build resilience and promote healing [19]. The simple act of identifying and drawing attention to children’s strengths can promote resilience and additionally serves to identify key family supports. Examples of incorporating a strengths-based approach in a clinical visit are shown in Table 1. Soliciting information about strengths also gives the practitioner insight regarding the youth’s sense of self and family dynamics. For example, if a youth struggles to think of anything they are proud of or good at, reinforcing positive parenting practices with parents or caregivers can help build resilience for that child. At times, especially for youth who have experienced trauma without the protective buffering of a caring adult, it may be difficult to identify strengths, and encouraging youth to recognize strengths such as listening skills, sports, reading, helping with childcare or household tasks, or even playing video games, can help re-frame their experiences to validate internal assets. It is important to acknowledge and validate hardships while also exploring and celebrating the youth’s role in overcoming adversity.

### 3.2. Create an Immigrant-Friendly Healthcare Environment

A critical component of TIC is preventing re-traumatization. Unfortunately, health care environments can be a source of trauma, racism, and xenophobia for immigrants. As a healthcare provider, one way to prevent re-traumatization is by advocating for safe and inclusive environments, including within your own clinic. The process of developing trust starts as soon as a patient interacts with the health care environment. Patients’ experiences in the waiting area, check-in desk, and rooming processes all present opportunities to feel welcome or rejected. An inclusive and immigrant-friendly healthcare environment requires regular and ongoing training for all staff that work in a clinic or hospital. The physical environment can also help to promote a sense of welcome. This can be done through the use of signs such as “All Are Welcome Here” posters or signage in multiple languages. Encouraging the hiring of providers and clinic staff who reflect the community being served is another important step in the creation of an inclusive environment [20].

Depending on your location of practice, you may have to consider safety planning in your clinic in the event of an immigration raid. For example, considering what information to document in the medical record about a patient’s legal or immigration status or what the clinic staff will do in the case of immigration police presence ahead of time can help to protect your patients [21]. Familiarity with legal requirements when working with immigration enforcement officers, such as articles from the National Immigration Law Center for health care providers in the United States [22], assist providers in knowing what information is protected to avoid unintentionally sharing information that may result in harm to patients or families.

### 3.3. Promote Trusting Relationships Within the Health Care Environment

One of the fundamental principles of TIC is the importance of trusting relationships and the knowledge that relationships are protective buffers against toxic stress. Primary care providers, in particular, have enormous potential to increase a patient’s sense of safety in their health care home. In addition to creating an immigrant friendly clinic, it is important to recognize an individual provider’s role in developing trust and rapport. One of the essential components of healing is the creation of safe spaces in which everyone, from parents to children and youth, feels welcome. Empathy and motivational interviewing are critically important tools in establishing this safety. Acknowledge the steps the family or youth have taken in entering into a new and unfamiliar system. The fact that a family has arrived in the exam room is a big step, often in spite of multiple barriers that families have to overcome even before we meet them. Triaging needs according to urgency can continue to develop trust and rapport. It is imperative to allow the patient and family to drive the agenda as much as possible. For example, if a youth is suffering from severe depression or anxiety, counseling about routine health care maintenance may be more effective if done after the patient’s mental health has been stabilized and urgent needs met. Of note, the model of trust and mutual respect as an equal partnership between patient and provider is a relatively new development and may be unfamiliar to patients and families. This model of care is a concept worth discussing to allow families and youth to understand that, in this model, they play an important role in making decisions about interventions and health care decisions.

The families of refugee and immigrant youth in your office likely have extensive experience with the health care system in their country of origin, which may be very different from the setting in which you are seeing the patient. The pathways for accessing care, the cost of care, and the methods for receiving results may be barriers for the receipt of health services. Knowing when to utilize an after-hours urgent care or emergency department rather than a primary care office is one example: this may be an unfamiliar model to many families, and education about the role of the primary care office may prevent unnecessary emergency department visits.

Health providers can help to mitigate some of these barriers by intentionally providing education about the healthcare system, including how to access primary care or what to do in case of an emergency, the role of specialists, and how to pick up medications. Seeking help from colleagues such as social workers, patient advocates, or support groups can be extremely useful in helping families navigate the new system [12,23].

### 3.4. Ask for Permission to Discuss Potentially Difficult Subjects

A trauma-informed approach acknowledges that youth and families can be re-traumatized if they are forced to prematurely share information about traumatic events. Therapists working with victims of trauma often refer to the “window of tolerance”: that is, the practice of sharing emotions within a tolerable range [24]. Exceeding the window of tolerance too quickly can result in unacceptable levels of emotional pain and is unlikely to be therapeutic. Borrowing from principles of motivational interviewing, we recommend asking patients and families for permission to discuss sensitive topics [25]. This places the youth in control of their own narrative and allows them to avoid sharing details until they are ready. Ideally, providers should share with the patient why they are asking for information, followed by a request to ask additional questions. For example, “I want to make sure that we’re giving you the best medical care possible. In order to make sure I understand your history, I would like to ask a few additional questions about what happened when you were apart from your family. Is that okay with you?”

A similar approach should be utilized with invasive physical examinations. For example, if a pelvic exam is necessary due to clinical concerns, explain the need for the exam and ask for the child or adolescent’s permission to perform the exam. Explain that the child can ask the examiner to stop at any time [25]. If a child is unable to tolerate or assent to a physical exam after the procedure has been explained in a trauma-informed approach, they may require sedation or alternate clinical care to prevent re-traumatization.

### 3.5. Recognize the Impact of Trauma on the Developing Brain, Various Manifestations of Trauma, and Screen for Trauma and Associated Mental Health Conditions

An important aspect of TIC is acknowledgement of the pervasive impact of trauma, including screening for past experiences of trauma. Depression, anxiety, behavioral issues, and PTSD are associated with trauma and have higher prevalence in immigrant and refugee youth than their counterparts who have not experienced similar processes of acculturation or discrimination [26,27,28]. Immigrant and refugee youth are also less likely than their peers to access health care and preventive services [29], increasing the likelihood of poor outcomes. Unfortunately, literature has shown that immigrant and minority youth are less likely to be screened for depression than their native-born or White counterparts [30]. Ensuring access to quality screening can promote access to effective treatments. Use validated tools for screening for depression and anxiety, such as the Patient Health Questionnare-9 (PHQ-9) [31], Generalized Anxiety Disorder 7-item scale (GAD) [32], or Screen for Child Anxiety-Related Emotional Disorders (SCARED) [33] tools. These tools also have evidence suggesting they are effective for diverse populations and in a variety of languages [34,35,36,37,38,39]. There are surveys that screen for post-traumatic stress disorder (PTSD), such as the abbreviated UCLA PTSD Reaction Index; however, children rarely meet the full criteria for PTSD, as the criteria has been derived from adult populations [40,41]. Whenever possible, administer surveys in the patient’s first language. Finally, screening for sleep problems is essential because of the detrimental effects of trauma on sleep patterns [42].

Additionally, acknowledge the role of previous coping strategies on child and adolescent behavior and development. Youth who have experienced trauma use coping mechanisms to manage chronic stress. These coping mechanisms may have even been adaptive and even necessary for survival in prior circumstances, especially for youth who have experienced extreme violence. However, under less life-threatening circumstances, the coping mechanisms that have served the youth well in the past may create difficulties in the new environment [43,44]. Due to these coping mechanisms, it is wise to consider trauma in the differential diagnosis of youth presenting with behavioral concerns [45,46]. Externalizing behaviors associated with trauma are frequently misdiagnosed as ADHD or ODD. Pharmacological treatment for ADHD or ODD may not be effective or appropriate for the treatment of trauma-related behavioral challenges; thus, it is important to screen for trauma in order to offer proper management and treatment. It can be difficult to differentiate the cognitive impairment associated with chronic stress, anxiety, or depression from ADHD [45]. Therefore, if a diagnosis is in doubt and treatments have been ineffective, referring for an in-depth psychiatric assessment can often provide clarity.

Note that if a family or youth shares stories of trauma, it is important to validate their experiences and emotions and take the necessary time to follow-up positive screens or shared stories.

The list below includes experiences of trauma that may be more prevalent in immigrant and refugee families.

Potential sources of trauma for immigrant and refugee youth

- Anxiety about the possibility of parental deportation or safety of family members in the country or origin;
- Family separation, either planned separation due to immigration logistics or separation as a result of immigration policy or detention;
- Bullying or victimization at school;
- Physical or sexual abuse;
- Dangerous conditions during migration;
- Family conflict or intrafamilial violence;
- Unsafe neighborhoods or gun violence (in country of origin and after relocation);
- Racism and microaggressions (both in country of origin and after relocation).

### 3.6. Treat Trauma-Related Disorders Appropriately

TIC requires treating trauma. While the evidence-based guidelines for the medical management of trauma and associated disorders are beyond the scope of this article, a brief summary of initial treatment for trauma and its symptoms is provided. Health promotion can be utilized to downregulate the stress response system, for example encouraging habits such as good sleep hygiene, nutritious eating, regular physical activity, play, and engaging with social support systems [41,46]. There is good evidence that trauma-focused cognitive behavioral therapy and child–parent psychotherapy are effective [41]. Medications for depression, anxiety, and sleep difficulty can be utilized as appropriate, and referrals made for care outside of the primary provider’s scope of practice. Ideally, the primary care office can serve as a behavioral health home with integrated mental health practitioners and “warm handoffs” to trusted providers [6,23]. When this is not possible, ensure that children are referred to trustworthy providers who have experience in TIC.

Note that diagnoses of depression or anxiety may be stigmatizing to patients, particularly in certain immigrant communities. Framing depression or anxiety as a result of trauma can sometimes help families better understand these disorders; for example, utilize the phrase “stress- and trauma-related disorder” to explain the root symptoms being treated. Qualifiers such as anxiety or depression can used as secondary diagnoses, with an explanation for youth and parents regarding how these symptoms are the result of “what happened to you” rather than “what’s wrong with you.”

In the treatment of trauma and related mental health disorders, foster agency in youth by asking about their treatment preferences. Whenever possible, give them options in choosing their care. If their preferred treatment option is safe, allow it to the extent possible. Ensure that patients know how to contact the clinic or seek follow-up or emergency care.

### 3.7. Utilize a Two-Generational Approach to Care 

When children and youth completed the transnational journey with most of the close family, remember that each individual of the family has experienced the same traumatic events (war, extreme poverty, persecution, etc.). Parents and caretakers can offer one of the most protective factors for children and adolescents in surviving stress and trauma and supportive and consistent relationships with a caring adult can buffer youth from the negative impacts of trauma [14,26,46]. Given the role parents and caretakers play in the management of childhood trauma, healthcare providers should extend their triage to caregivers. Support caregivers in navigating a new and unfamiliar health care system in order to ensure that they are able to access the same care the youth is receiving by working with ancillary services, such as social workers or community mental health practitioners.

Youth and children often lose the adult protection that caregivers provided in their country of origin. Children and youth experience “adultification” as they learn the new rules of a culture and master language skills more quickly than their caregivers [47,48]. Children then run the risk of becoming the internal cultural brokers of the family. Providers need to be aware of these dynamics and recognize the anxiety it can cause in youth. Depression may also result from feelings of loss of parental efficacy that parents and caregivers may experience. For example, parents and caregivers may have been stripped of the privileges afforded to them in their country of origin (such as fluency in the primary language, professional status, or primary breadwinner). These losses can have significant effects on parental or caregiver mental health and, by extension, on the health of children and adolescents.

Immigrant and refugee parents often need more coaching during their child’s adolescence than would have been necessary if they were living in their country of origin. As these parents did not complete the majority of their developmental growth in the host country, they can feel lost or disoriented when guiding their adolescence through their teenage years in an unfamiliar environment. Additionally, the new culture may foster more individualistic behaviors than the country of origin, which can result in a clash of values between an adolescent seeking independence and parents seeking to protect their children and promote community-oriented values [48].

### 3.8. Know Your Own Local Resources and Make Sure They Are Trustworthy

Partnerships with other individuals and agencies in your area outside of health care can be an invaluable resource for TIC with refugee and immigrant youth. Immigrant and refugee youth and their families may have past experiences of trauma that impact their trust or engagement with various services and may be unfamiliar with how to access services in an unfamiliar environment. Healthcare providers may be the first professionals to interact with immigrants and refugee youth and families and as such can play a vital role in bridging interrelated systems and services. These can include experts in your own country’s immigration law, counselors and psychologists practicing trauma-informed mental health care in the youth or caregivers’ first language, community centers for community building and physical activity, and resources for nutritious food and safe housing. Legal assistance in particular can be helpful if immigrants are undocumented [9,12]. Children and adolescents who live with undocumented family members have been shown to have high anxiety levels about the fear of deportation of family members [49,50], and lack of documentation can result in additional barriers to care that result in poorer health outcomes [51,52,53]. Thus, it is important to refer to low-cost or free legal services to promote health for the entire family.

### 3.9. Recognize that Trauma May Not End after Migration

In an ideal world, all youth on the move would not be exposed to new trauma once the migration journey is complete. However, it is important to acknowledge that trauma in the form of racism, bias, and microaggressions may continue long after migration [4,28,54]. Chronic exposure to racism has been associated with negative long-term health outcomes [20,55] and with perceived poor quality of medical care [4]. This relationship can be explored with your patients using the principles of TIC. As most youth will spend a majority of their time in educational environments, it is important to screen for bullying, racism, and victimization at school. Finally, this can be an opportunity to explore the youth’s resilience mechanisms, emphasizing the unique cultural strengths of the individual and family, including conversations about racism and cultural socialization.

### 3.10. Advocate for Your Patients Both in and outside the Clinic: For Your Patients and for Yourself

Using principles of TIC can provide safety for families and promote recovery, but it is also important to recognize that the act of caring for traumatized youth can be difficult and may lead to secondary traumatic stress. For health care providers on the front line of listening to patients’ stories, community advocacy is a strategy to transform secondary trauma, grief, and anger into action. Healthcare providers are well respected members of society in many communities and as such have unique privileges and power and are also uniquely positioned to listen to their patients’ stories directly. Using this power and knowledge to push for immigrant-friendly policies, a diverse healthcare workforce, and anti-racist policies can allow for health care providers to harness emotions to action. Healthcare providers are in an ideal position to write editorials for local and national news outlets, write letters to governing representatives, testify before local and national legislators, and organize within professional governing bodies to advocate for best practices for the care of immigrant and refugee families.

## 4. Conclusions

This discussion of TIC for refugee and immigrant youth is far from exhaustive. However, these clinical pearls can provide a concrete framework for incorporating a trauma-informed lens to your practice, including in relationship-building, diagnosis, management, and promotion of resilience. Utilizing the principles of TIC can also allow for providers to both recognize and treat experiences of trauma while also seeing beyond the trauma and develop an appreciation for the unique strengths of each patient and family. In turn, applying TIC can help promote well-being and health not just for immigrant and refugee youth and families, but also within our health care system by challenging and improving health care delivery from a trauma-informed perspective more broadly.

### Ten Clinical Pearls of Trauma Informed Care for Refugee and Immigrant Youth

1. Practice a strengths-based approach to care;
2. Create an immigrant-friendly healthcare environment;
3. Promote trusting relationships within the healthcare environment;
4. Ask for permission to discuss potentially difficult subjects;
5. Recognize the impact of trauma on the developing brain and various manifestations of trauma and screen for trauma and associated mental health conditions;
6. Treat trauma and its associated symptoms appropriately;
7. Utilize a two-generational approach to care;
8. Know your own local resources and make sure they are trustworthy;
9. Recognize that trauma may not end after migration;
10. Advocate for your patients both in and outside the clinic: for your patients and for yourself.

## Figures and Tables

**Table 1:** Strengths-based approach to care: clinical examples.

Clinical Skill	Example
Lead the social history or psychosocial assessment with questions about family and patient strengths	“Tell me a little bit about yourself. What are some things that you’re really proud of?” “What is something you’re good at?”
Gather information about family supports, and strengthen those relationships when possible	“If something difficult were to happen, who would be available to help?” “If something really good were to happen, who would be cheering for you?”
Congratulate patients and families on progress or accomplishments	“I’m so glad to hear that you are smoking fewer cigarettes—that’s wonderful! That’s a really challenging task. I can tell that you really care about your kids and are motivated to get their asthma under control. You should be really proud of your hard work.”
Acknowledge specific strengths, without stereotyping or making assumptions about religious, ethnic, or cultural groups	“That’s pretty great that you speak both English and Spanish. It’s a huge advantage when looking for jobs or applying to college—make sure to put that on all your applications.” “It sounds like your extended family is very close. I’m glad you have so much support available—it’s really important when taking care of your children and yourself. You should be proud of all the effort you’ve put into keeping those relationships strong.”
Help patients and families build on past success to continue to build resilience	“It sounds like it was really challenging to cut out soda for the whole family, but you’ve done it for a whole month now! That is really going to set a healthy example for your kids. What would be another step that you could take as a family to help Dad manage his diabetes?”
